# Consequences of obesity in outcomes after cardiac surgery: analysis of the ARIAM registry

**DOI:** 10.1186/cc12171

**Published:** 2013-03-19

**Authors:** EC Curiel-Balsera, J Muñoz-Bono, MJ Delgado-Amaya, R Hinojosa-Pérez, A Reina-Toral, A Gordillo-Brenes, R Rivera-Fernández

**Affiliations:** 1 Hospital Regional Carlos Haya, Málaga, Spain; 2 Virgen del Rocio Hospital, Seville, Spain; 3 Virgen de las Nieves Hospital, Granada, Spain; 4 Puerta del mar Hospital, Cádiz, Spain

## Introduction

Obesity is a disease that affects a large part of the population and has been associated with worse outcomes after cardiac surgery. The aim of our study is to evaluate the consequences of obesity related to postoperative complications, hospital length of stay and mortality.

## Methods

An observational, prospective, multicenter study of patients included in the ARIAM registry of adult cardiac surgery between March 2008 and March 2011. We analyzed clinical variables, the surgical procedure, postoperative complications and mortality, comparing the group of patients with body mass index (BMI) greater or less than 30 kg/m^2^.

## Results

The study included 4,712 patients with a mean age of 64.03 (SD ±12.08) years, BMI 28.53 (SD ±4.7) and EuroSCORE 5.58 (SD ±2.91). In 1,940 patients (35.7%) BMI was >30 kg/m^2^. There were no differences in the development of overall postoperative complications (33% in obese and 35.8% in nonobese, *P *= 0.07), although less appreciated were reoperation rate or stroke, as well as further development of postoperative renal failure. After adjusting for severity and length of cardio bypass time, obese patients had lower mortality without being statistically significant, OR = 0.94 (0.79 to 1.04). There were no differences in ICU length of stay, but obese patients had greater ward length of stay, 9.04 (10.43) versus 1.18 (9.2) days, *P *= 0.01.

## Conclusion

Obese patients undergoing cardiac surgery have mortality, rate of complications and length of stay similar to nonobese patients. Obese patients required less reoperation but developed more frequent postoperative renal failure.
